# Supplementary material for: Local Immune Activation and Age Impact on Humoral Immunity in Mice, with a Focus on IgG Sialylation
Source: Vaccines (Basel). 2024 Apr 29;12(5):479. doi: 10.3390/vaccines12050479 (PMC11125885; doi:10.3390/vaccines12050479)
Supplement: Supplementary file 1 [file vaccines-12-00479-s001.zip › vaccines-2953621-supplementary.pdf]

Figure S1

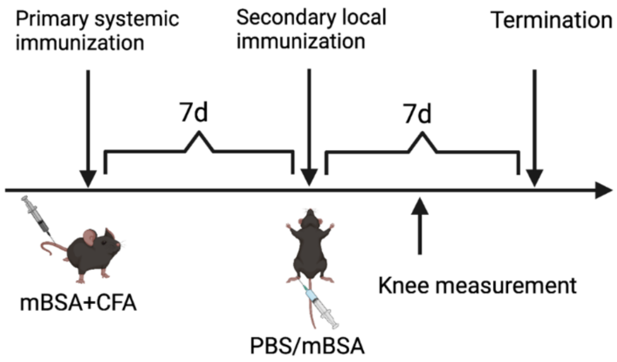

Figure S1: Experimental scheme of antigen-induced arthritis (AIA) mouse model. The mice were immunized by intradermal injection of methylated bovine serum albumin (mBSA) emulsified in complete Freund's adjuvant (CFA), followed seven days later by an intra-articular injection of mBSA in knee joints. Control mice received intra-articular PBS injection in the knee joint. Seven days after the IA injection experiment was terminated.

Supplementary Figure 2

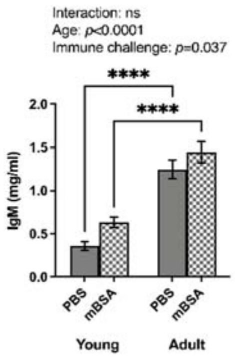

Figure S2: The level of IgM is age-dependent rather than immune induction by mBSA. Serum was collected at termination on day 14, and the IgM (mg/ml) level was measured. Statistical evaluations were performed using a two-way analysis of variance (2way ANOVA) to evaluate the effect of age, the effect of immune challenge and the interaction between age and immune challenge. This was followed by Fisher's LSD multiple comparisons to calculate statistical differences between individual groups.

Table S1: Detected glycoforms in murine IgG2b (Uniprot entry P01867, peptide sequence EDYNSTIR) by LC-MS mass spectrometry analysis.

Glycoform composition[a]	Depiction[b]	Relative % intensity			
A-galactosylated (G0)		**Young**		**Adult**	
		**PBS**	**m-BSA**	**PBS**	**m-BSA**
**N1**	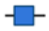	0.15 ±0.01	0.06 ±0.03	0.07± 0.01$^{\#}$	0.04±0.02
**H2N3F**	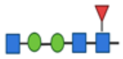	0.56 ±0.10	0.35 ±0.03*	0.32±0.02$^{\#}$	0.23±0.06
**H3N3F**	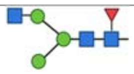	1.86±0.12	1.90 ±0.08	1.82±0.03	1.74 ±0.06
**H3N4F**	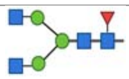	11.89±0.79	8.94 ± 0.52**	9.76 ± 0.64$^{\#}$	8.56±0.79
**H4N3F**	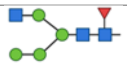	1.25 ±0.12	1.73 ±0.04	2.02±0.19$^{\#}$	2.63 ±0.33*$^{\#\#}$
Galactosylated (G1/G2)					
**H4N4**	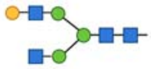	0.00±0.00	0.09±0.02	0.00±0.00	0.41±0.07***$^{\#\#\#}$
**H4N4F**	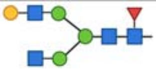	47.1±2.67	51.06 ±0.51	49.76±0.83	48.03±1.71
**H5N4**	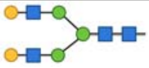	0.00±0.00	0.02±0.00	0.01 ±0.00	0.06 ± 0.03*
**H5N4F**	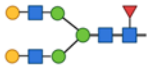	13.67±0.91	18.38±0.29***	17.03±0.71$^{\#\#}$	19.33± 0.68*
Sialylated (G1S/G2S)					
**H4N3FG1**	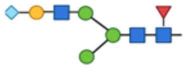	0.07±0.01	0.12±0.01	0.29±0.01$^{\#\#\#}$	0.36 ±0.05$^{\#\#\#}$
**H4N4FG1**	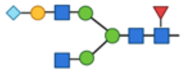	4.90±0.47	3.50±0.22*	4.57±0.31	3.57±0.32
**H5N4FG1**	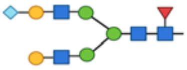	11.09±1.56	8.77±0.53	10.02 ±0.72	9.21 ±0.84
**H5N4FG2**	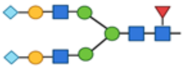	3.92±0.28	2.43±0.14***	2.59±0.06$^{\#\#\#}$	1.63±0.25**$^{\#}$

[a]H: hexose, N: N-acetyl hexosamine, F: fucose, G: N-glycolylneuraminic acid. [b]Symbols used: green circle: mannose, yellow circle: galactose, blue square: N-acetylglucosamine, red triangle: fucose, blue diamond: N-glycolylneuraminic acid (Neu5Gc). *Represents the significant difference between PBS to mBSA. #Represents the significant difference between young to adults. The proposed glycan structures are based on fragmentation analysis and literature. Statistical analysis was performed with the Fisher LSD test. Error bar denotes ± SEM.

Table S2: Detected glycoforms in murine IgG3 (Uniprot entry P03897, peptide sequence EAQYNSTFR) by LC-MS mass spectrometry analysis.

**Glycoform composition**[a]	**Depiction**[b]	**Relative % intensity**			
A-galactosylated (G0)		**Young**		**Adult**	
		**PBS**	**m-BSA**	**PBS**	**m-BSA**
**H2N3F**	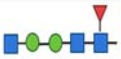	0.85±0.12	0.44±0.13	0.98±0.26	0.44±0.22
**H3N3F**	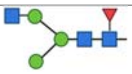	3.21±0.11	3.15±0.37	3.12±0.67	2.25±0.95
**H3N4F**	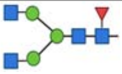	40.09±1.54	36.43±1.20	42.72±3.58	46.93±1.66$^{\#\#}$
Galactosylated (G1/G2)					
**H4N3F**	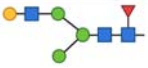	0.31± 0.17	0.00±0.00	0.33±0.19	0.26±0.17
**H4N4F**	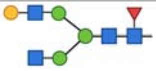	33.46±1.39	39.80±1.17*	36.33±1.96	39.70±1.69
**H5N4F**	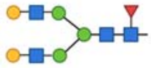	10.07±1.03	13.95±0.87	12.16±2.10	4.54±1.58**$^{\#\#\#}$
Sialylated (G1S/G2S)					
**H4N4FG1**	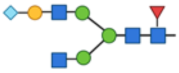	6.98±0.78	2.78±0.44***	1.44±0.37$^{\#\#\#}$	1.86±0.72
**H4N5FG1**	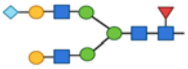	4.13±0.39	3.46±0.48	2.92±0.69	3.99±2.19
**H5N4FG2**	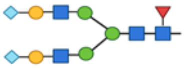	0.89± 0.35	0.00± 0.00**	0.00± 0.00$^{\#\#}$	0.00±0.00

[a]H: hexose, N: N-acetyl hexosamine, F: fucose, G: N-glycolylneuraminic acid. [b]Symbols used: green circle: mannose, yellow circle: galactose, blue square: N-acetylglucosamine, red triangle: fucose, blue diamond: N-glycolylneuraminic acid (Neu5Gc). *Represents the significant difference between PBS to mBSA. #Represents the significant difference between young to adults. The proposed glycan structures are based on fragmentation analysis and literature. Statistical analysis was performed with the Fisher LSD test. Error bar denotes ± SEM.
